# Suppressive Effect of Bioactive Extracts of *Bacillus* sp. H8-1 and *Bacillus* sp. K203 on Tomato Wilt Caused by *Clavibacter michiganensis* subsp. *michiganensis*

**DOI:** 10.3390/microorganisms10020403

**Published:** 2022-02-09

**Authors:** Hwajin Jang, Sang Tae Kim, Mee Kyung Sang

**Affiliations:** 1 Division of Agricultural Microbiology, National Institute of Agricultural Sciences, Rural Development Administration, Wanju 55365, Korea; hjjang@gmail.com (H.J.); stkim9312@gmail.com (S.T.K.); 2 Department of Applied Bioscience, Dong-A University, Busan 49315, Korea

**Keywords:** *Bacillus* spp. biocontrol, bioactive extract, *Clavibacter michiganensis* subsp. *michiganensis*

## Abstract

Tomatoes are cultivated worldwide, and are economically important. *Clavibacter michiganensis* subsp. *michiganensis* (*Cmm*) is a pathogen that causes canker and wilting in tomatoes, resulting in serious damage to tomato plants. We aimed to control *Cmm* proliferation using substances produced by useful microorganisms. The water extracts of strains H8-1 and K203 inhibited wilting caused by *Cmm* and slowed the pathogenic colonization in tomato plants. The relative expressions of *celA, celB, pat1,* and *pelA* of *Cmm* treated with the bacterial water extracts were reduced by 0.41-, 0.01-, 0.15-, and 0.14-fold for H8-1, respectively, and 0.45-, 0.02-, 0.13-, and 0.13-fold for K203, respectively, compared to controls at 72 h after treatments. In tomato plants inoculated with *Cmm*, when water extracts of H8-1 and K203 were treated, relative expression of *ACO* encoding 1-aminocyclopropane-1-carboxylic acid oxidase was suppressed by 0.26- and 0.23-fold, respectively, while *PR1a* was increased by 1.94- and 2.94-fold, respectively; PI2 expression was increased by 3.27-fold in water extract of H8-1-treated plants. As antioxidant enzymes of plants inoculated with *Cmm*, peroxidase and glutathione peroxidase levels were increased in K203-water-extract-treated plants, and catalase was increased in the case of the H8-1 water extract at 10 days after inoculation. In terms of soil enzyme activity, each water extract tended to increase urease activity and microbial diversity; in addition, K203 water extract increased plant growth. Thus, H8-1 and K203 water extracts can be used as potential biocontrol agents against *Cmm.*

## 1. Introduction

Tomatoes are the second most important crop in the world, with a total output of 182 million tons [1,2]. *Clavibacter michiganensis* subsp. *michiganensis* (*Cmm*) is a soil-borne bacterial pathogen that reduces tomato yield and quality, resulting in significant economic losses [3,4,5,6]. Most soil pathogens are Gram-negative, whereas *Cmm* is a Gram-positive bacterium belonging to *Actinomyces* that causes significant crop losses by causing wilt and canker disease in tomato plants [7,8,9,10]. *Cmm* systemically infects host plants through wounds, pores, and seeds, causing wilt and canker symptoms [7]. When *Cmm* infects the early stages of host plants, they develop systemic infections, referred to as primary infections, which affect the quality and harvest of fruits, and generally cause death. If *Cmm* infects older plants, they usually develop foliage infections referred to as secondary infections, which cause chlorosis of leaves, but may or may not affect the quality and yield of current crops [11]. During infection, colonization, and disease progression, *Cmm* secretes cell-wall-degrading enzymes such as cellulase, xylanase, and pectate degradation enzymes, which participate in the decomposition of plant cell wall components such as cellulose, xylan, and pectin, respectively, to promote *Cmm* colonization and nutrient acquisition [12].

About 16% of differentially expressed genes at 8 days post-inoculation are involved in defense during *Cmm* infection; defense-related genes result in the production and elimination of free oxygen radicals, enhancement of protein turnover, and hormone (including ethylene) synthesis and reaction [13]. Moreover, in order to combat pathogen infections, host plants activate basic defense responses through pathogenesis-related (PR) proteins when recognizing extracellular pathogen-related molecular patterns (PAMPs) [14,15]. In general, these PR proteins have antibacterial properties, and are involved in cellular activities such as defensive signaling, cell wall hydrolysis, production of active oxygen species, contact toxicity, and alkalization of the medium [16,17]. It is also well known that the plant hormones jasmonic acid (JA), salicylic acid (SA), abscisic acid (ABA), and ethylene (ET) act as dominant primary signals in the regulation of plants’ local and systemic defense responses [18]. In general, pathogen-induced systemic acquired resistance (SAR) depends on the SA-regulated signaling pathway [19], and induced systemic resistance (ISR) by beneficial microorganisms generally relies on JA signaling [20,21,22].

Despite this defense, many plants are infected, and several approaches for disease management have been developed; for instance, various chemical pesticides are used for disease control [23]. Despite the clear positive contributions to the efficient control of plant fungal diseases and pests, there is a lack of effective pesticides for controlling bacterial pathogens in rapidly developed infection [24]. In addition, concerns are growing over the side effects of chemical pesticide abuse in terms of soil and water pollution, as well as toxicity to beneficial organisms [25,26]. Accordingly, the increase in the demand for pesticide substitutes provides opportunities for the expansion of biological control [27,28]. Amkraz et al. [29] reported biological control agents with antagonism against *Cmm* using fluorescent pseudomonades under greenhouse conditions. In addition, a study by Abo-Elyousr et al. [30] found that *Bacillus subtilis*, *B. amyloliquefaciens*, *Pseudomonas fluorescens*, and *P. aeruginosa* reduced the disease severity caused by *Cmm*, and it was confirmed that the four bacteria produced bioactive metabolites such as siderophores, HCN, and indole acetic acid, which could be applied as eco-friendly alternatives in the future.

Our study aimed to find effective bacteria-derived extracts as eco-friendly alternatives for controlling *Cmm* in vitro and *in planta*. We also investigated the mode of action of the bacterial extracts from various perspectives including *Cmm* colonization and pathogenicity-related gene expression, plant antioxidant response and gene expression, and soil activities, by studying total microbial activity, phosphatase and urease activities, and diversity, based on the use of 31 carbon sources. The results of this study allowed us to understand the effects of bacterial bioactive extracts on the control of *Cmm* in broad contexts of *Cmm* infection stages, host plant response, and soil microbial environments.

## 2. Materials and Methods

### 2.1. Bacterial Strains

In total, 95 epiphytic strains were isolated from the leaves, stems, flowers, and rhizospheres of various plants (tomatoes, cucumber cabbages, turmeric, chives, and strawberries) in various regions (Miryang, Kimje, Gangneung, and Jeju). Samples (1 g) were added to 9 mL of sterile 10 mM MgSO_4_ solution, incubated for 30 min at 160 rpm and 28 °C, and smeared on tryptic soy agar (TSA, Difco, Sparks, MD, USA) medium containing cycloheximide (50 μg/mL) to prevent fungal growth and obtain only bacteria. After three days of incubation, morphologically distinct colonies were isolated and stored in tryptic soy broth medium (TSB, Difco) supplemented with 20% glycerol at −80 °C before use [31].

### 2.2. Cell Viability Test and Cellulase Test

All of the isolated strains were cultivated on TSA for 3 days at 28 °C, and single colonies were transferred to 5 mL of TSB. After incubation for 2 days at 160 rpm and 28 °C, bacterial cells were removed by centrifugation at 6000 rpm for 20 min, followed by filtering (0.22 um syringe filter, Techno Plastic Products AG, Product NO 99722, Trasadingen, Switzerland), and the cell-free broth was placed at 4 °C before use. The bacterial pathogen, *Clavibacter michiganensis* subsp. *michiganensis* (*Cmm*, KACC 16995), was grown on nutrient broth yeast extract agar (NBYA) medium (8 g of nutrient broth, 2 g of yeast extract, 2.5 g of glucose, 2 g of KH_2_PO_4_, 0.5 g of KH_2_PO_4_, 1.5 g of MgSO_4_·7H_2_O, and 15 g of agar per liter) at 28 °C for 3 days [32], and a single colony was propagated in nutrient broth yeast extract broth (NBY) at 28 °C and 160 rpm for 24 h. *Cmm* pellets were obtained by centrifugation at 3500 rpm for 5 min twice [33], and *Cmm* suspension (OD_640_ = 1.0) was prepared in 10 mM MgSO_4_ solution. The mixture of a cell-free supernatant and *Cmm* suspension (9:1, *v*/*v*) was incubated in each well of 96-well plates for 24 h; PrestoBlue Cell Viability Reagent (10%, *v*/*v*, Invitrogen, Waltham, MA, USA) was added to the 96-well plates and incubated at 37 °C for 10 min. Cell viability was measured at a wavelength of 570 nm using a microplate reader (Infinite M200 Pro, TECAN, Männedorf, Switzerland), and compared with that of a mixture of *Cmm* suspension and TSB.

For measurement of the cellulase activity of *Cmm*, M9 minimal medium (7 g of Na_2_HPO_4_·7H_2_O, 3 g of KH_2_PO_4_, 0.5 g of NaCl, 1 g NH_4_Cl, 0.492 g of MgSO_4_·7H_2_O 0.111 g of CaCl_2_ and 15 g of agar per liter) containing 0.1% *w*/*v* of yeast extract and 0.5% *w*/*v* of carboxymethyl cellulose (CMC) was used. The mixture (10 μL) of 1/10 diluted cell-free supernatant and *Cmm* suspension (9:1, *v*/*v*) was incubated in the center of M9 minimal medium for 3 days at 28 °C, and then stained with Congo red for 2 h. After washing three times with 1 M NaCl, the yellow color was recorded as a positive response [34].

### 2.3. Plant Material and Inoculation

Tomatoes (*Solanum lycopersicum*, ‘Superdotaerang’, Koregon, Anseong, Korea) were used for all plant experiments under greenhouse conditions, with a 16/8 h (light/dark) photoperiod at 25 ± 5 °C and 50 ± 5% relative humidity. Tomato seeds were grown in pots (10 cm, diameter) containing a potting mixture (Baroker, SeoulBio, Eumseong, Korea). *C*. *michiganensis* subsp. *michiganensis* was cultured as described above. For the inoculation of *Cmm* in tomato (five-to-six-leaf stage) plants [23], four-holes (1.5 cm distance to plants, 5 cm depth) were prepared in all tested plants, including controls, for uniform disease occurrence, and then the mixture (20 mL/plant, 1:1, *v*/*v*) of *Cmm* suspension (10^9^ CFU/g) and tenfold-diluted supernatant was put into the four-holes (5 mL/hole). A mixture of *Cmm* suspension and tenfold-diluted TSB, and streptomycin (250 µg/g of soil)—which acts against *Cmm*—were used as the negative and positive controls, respectively. For the plant growth promotion test, tomato plants (five-to-six-leaf stage) were used. Water extracts of H8-1 and K203 (1000 µg/g of soil) were added; three weeks later, the weight of the upper part of the plants was measured. The experiment was conducted in three replicates of eight plants each.

### 2.4. Bacterial Identification and Characterization

To identify the three strains (K203, H8-1, and GLSH03), total genomic DNA was extracted, and 16s rRNA was amplified using primers 785F and 907R, and compared with sequences of type strains using the EzBioCloud database. Phylogenetic trees were constructed using the neighbor-joining method of the Molecular Evolutionary Genetics Analysis (MEGA) program. API 50CH/B and API ZYM (bioMérieux, Marcy-l’Étoile, France) were used for bacterial characterization. Three strains—K203, H8-1, and GLSH03—were adjusted to OD_600_ = 0.45 for API 50CH/B and OD_600_ = 0.67 for API ZYM, and the bacterial suspension was added to each strip according to the manufacturer’s instructions. API 50CH/B and API ZYM were read at 48 h and 4 h after incubation at 28 °C, respectively.

### 2.5. Preparation of Bacterial Extract and Biocontrol Activity in Tomato Plants

Three strains—K203, H8-1, and GLSH03—were cultured, and cell-free supernatants were prepared as described above. A cell-free supernatant was sequentially partitioned by solvents, n-hexene, dichloromethane, ethyl acetate, and n-butanol based on polarity. Each mixture of solvent and cell-free supernatant (1:1, *v*/*v*) was shaken at 200 rpm for 12 h, and each organic solvent and water fraction was collected. The collected fractions were evaporated and concentrated. The final solvent and water fractions were dissolved in methanol and HPLC-grade water, respectively [35]. The concentrated extracts were stored at 4 °C after filtering (0.22 μm syringe filter) before use. To evaluate the effect of each extract on *Cmm* viability, various concentrations of solvent extracts (0, 1, 10, 50, 100, 500, and 1000 µg/mL) and water extracts (0, 1, 10, 25, 50, and 100 mg/mL) were tested. After determining the bioactive extract via the *Cmm* viability assay, a plant test was conducted as mentioned above. The solvent extracts did not suppress *Cmm* viability (data not shown). Various concentrations (1, 10, 100, and 1,000 μg/g of soil) of the water extract and *Cmm* suspension (10^9^ CFU/g of soil) were added into four holes (5 cm depth) at a distance of 1.5 cm from tomato plants, and controls were also treated in the holes. Twenty days after inoculation, disease incidence, severity, and area under the disease progress curve (AUDPC) were evaluated. Disease severity was scored as follows: 0 = no symptoms; 1 = 0–25% leaf wilting; 2 = 26–50% leaf wilting; 3 = 51–75% leaf wilting; 4 = 76–100% leaf wilting; and 5 = dead [36].

### 2.6. Cmm Colonization in Tomato Plants

As described above, *Cmm*-inoculated tomato plants (five-to-six-leaf stage) were prepared, and tomato nodes (1 to 4) from the soil line were sampled at 1, 3, 7, 15, and 20 days after inoculation. Sampled stem segments were homogenized using a mixer mill (Retsch, MM200), followed by smearing serial dilutions on the bacterial canker of the tomato (BCT) medium [37]. Subsequently, the number of CFUs was counted for quantification of the *Cmm* population in tomato plants. The experiment was conducted twice, with five replicates each.

### 2.7. Antioxidant Enzyme Assay

For the antioxidant enzyme assay, tomato leaves were collected 1, 3, 5, 7, and 10 days after inoculation and homogenized using a mixer mill (Retsch, MM200, Haan, Germany) in liquid N_2_. For the catalase assay, sampled leaves were homogenized in 250 µL of 50 mM potassium phosphate buffer containing 1 mM EDTA, the supernatant was collected after centrifugation at 10,000 rpm for 15 min at 4 °C, and the activity was determined using a catalase assay kit (Cayman, Item No. 70700, Ann Arbor, MI, USA). For superoxide dismutase activity, the leaves were homogenized in 500 µL of 20 mM HEPES buffer containing 1 mM EGTA, 210 mM mannitol, and 70 mM sucrose, and then centrifuged at 10,000 rpm for 15 min at 4 °C, and superoxide dismutase activity was determined using a superoxide dismutase assay kit (Cayman, Item No. 706002, Ann Arbor, MI, USA). For glutathione peroxidase activity, leaves were homogenized in 1 mL of homogenization buffer containing 50 mM Tris-HCl (pH 7.5), 5 mM EDTA, and 10 mM DTT, and then centrifuged at 10,000 rpm for 15 min at 4 °C; glutathione peroxidase activity was measured using a glutathione peroxidase assay kit (Cayman, Item No. 703102, Ann Arbor, MI, USA). Peroxidase activity was assayed following the manufacturer’s instructions (Abcam, ab155895, Cambridge, UK). The total protein content of each sample was determined using the Bradford method [38]. The experiment was conducted twice, with five replicates each.

### 2.8. Pathogenicity-Related Gene Expression of Cmm

*Cmm* was incubated in M9 minimal medium (MM) containing 0.4% CMC at 28 °C and sampled at 0, 24, 48, and 72 h after incubation for *Cmm* RNA extraction. Bacterial cells were collected by centrifugation at 13,000 rpm for 1 min at 4 °C, and the pellet was frozen and stored at −80 °C. Bacterial RNA was extracted using an easy-spin (DNA-free) total RNA extraction kit (Intron Biotechnology Catalog 17221, Seongnam, Korea). RNA quantification was performed using a NanoDrop spectrophotometer (Thermo Fisher Scientific, Waltham, MA, USA). cDNA was synthesized using PrimeScript^TM^ RT Master Mix (Takara, Catalog #RR036A, Kusatsu, Japan) for 15 min at 37 °C and 15 s at 85 °C. Specific primers were used for pathogenicity-related gene expression, as described in Table 1. The *Cmm* gene *gyrA* was used as the reference gene. The real-time qPCR reaction was conducted with 3 µL of cDNA, 1 µL of 10 pmol of each primer, 10 µL of SYBR Green with high ROX (Enzynomics, Catalog RT500S, Daejeon, Korea), and 5 µL of RNase-free water, using a qPCR CFX 96^TM^ Real-Time System (Bio-Rad, Hercules, CA, USA). The cycling program consisted of an initial denaturation step of 10 min at 95 °C, followed by 40 cycles at 95 °C for 5 s, 60 °C for 15 s, and 72 °C for 20 s [39]. The experiment was conducted twice, with four replicates each.

### 2.9. qRT-PCR for Plant Gene Expression

Tomato samples (leaves) were collected at 0, 24, 48, and 72 h after inoculation in tomato plants and homogenized using a mixer mill in liquid N_2_. For qRT-PCR analysis, total RNA was extracted using a plant RNA extraction kit (Intron, Catalog 17491, Seongnam, Korea), and the RNA was quantified using a NanoDrop spectrophotometer. cDNA was synthesized using PrimeScript^TM^ RT Master Mix for 15 min at 37 °C and 15 s at 85 °C. For plant gene expression, specific primers were used, as described in Table 2. The tomato plant gene *GAPDH* was used as the reference gene. The real-time qPCR reaction was conducted with 2 µL of cDNA, 1 uL of 10 pmol of each primer, 10 µL of SYBR Green with high ROX, and 6 µL of RNase-free water, using the qPCR CFX 96^TM^ Real-Time System. The program used for qRT-PCR was 10 min at 95 °C (initial denaturation), followed by 40 cycles of 15 s at 95 °C, 20 s at 52 °C, and 20 s at 72 °C [40]. The experiment was conducted twice with four replicates each.

### 2.10. Soil Microbial Activities and Diversity by EcoPlate

Soil samples were collected at 5, 10, 15, and 20 days after inoculation of tomato plants. The experiment was conducted twice, with five replicates each. For total microbial activity, fluorescein diacetate hydrolysis (FDase) of soil samples was measured as described by Schnürer and Rosswall [41]. Soils (1 g) were added to 4 mL of 60 mM sodium phosphatase buffer (pH 7.6), fluorescein diacetate (FDA, final concentration 10 µg/mL), and 4 mL of 60 mM sodium phosphate buffer, and the mixture was incubated at 25 °C for 1 h. By adding acetone, the reaction was terminated and filtered through a two-layer filter paper (Whatman, No. 2, Maidstone, UK). The filtered solution was then measured at OD 490 nm. The soil urease activity was determined according to the method described by Kandeler and Gerber [42]. Soils (1 g) were added to 0.5 mL of 0.72 M urea solution and 4 mL of 0.06 M borate buffer at pH 10.0, and incubated at 37 °C for 2 h. Finally, 1 N KCl was added to 0.01 N HCl for termination. After incubation for 30 min, the soil suspension was filtered using a two-layer filter paper (Whatman No. 2, Maidstone, UK). Na salicylate solution (1 mL) and 0.1% Na dichloroisocyanurate (0.4 mL) were added to the filtrate (1 mL); after incubation for 30 min, the enzyme activity was determined at 690 nm.

Soil acid phosphatase activity was determined using the method described by Tabatabai and Bremner [43]. Soils (1 g) were added to the modified universal buffer (MUB, 4 mL), toluene (0.25 mL), and p-nitrophenyl phosphate (PNP, 1 mL) solution, and incubated at 37 °C for 1 h. Calcium chloride (0.5 M) and sodium hydroxide were added to the soil suspensions, filtered using a two-layer filter paper (Whatman, No. 2, Maidstone, UK), and measured at OD 420 nm. For the EcoPlate assay, soil samples were prepared according to the manufacturer’s instructions (Biolog, Catalog 1506, Hayward, CA, USA), and EcoPlates were measured every 24 h for five days using a microplate reader (Infinite M200 Pro, TECAN).

Shannon diversity index was calculated formula as follows [44]:

Shannon diversity index: ∑P_i_ × (ln P_i_)

Pi: the OD 590 value for i divided by the mean OD 590 value of the 31 wells.

### 2.11. Statistical Analyses

Statistical analysis of the data was conducted using Statistical Analysis Systems (SAS Institute, Cary, NC, USA). For the analysis of ordinal data, nonparametric analysis was used based on the ranks of the data, while percentage data were statistically analyzed after arcsine square root transformation. All data from repeated experiments were pooled after checking the homogeneity of variances with Levene’s test and performing further statistical analyses. Analysis of variance was performed using general linear model procedures, and means were separated using the least significant difference (LSD). AUDPC was determined using the formula described by Shaner and Finney [45]: AUDPC = (X_i+1_+ X_i_)(t_i+1_ − t_i_)/2, where X_i_ is the disease severity or incidence at the ith observation, t_i_ is the time (day) at the ith observation, and n is the total number of observations. All results are shown as the mean ± standard error.

## 3. Results

### 3.1. Cell Viability and Cellulase Test

A total of 95 bacterial strains were isolated from various parts of vegetables, and cell-free supernatants were used in this study. A total of 27 out of the 95 bacterial supernatants were pre-selected based on more than 50% relative reduction in *Cmm* viability, and 42 supernatants contained cellulase activity on CMC media (Table 3). Among the 42 supernatants, only 7—strains GLSH03, H2-7, H8-1, HN05, HN12, K203, and TS3-1—had activities responsible for both the inhibition of *Cmm* viability and cellulase activity.

### 3.2. Disease Suppressive Activity

Among the seven bacterial supernatants, GLSH03, H8-1, and K203 supernatants significantly (*p* < 0.05) suppressed disease incidence compared to water and 1/10-diluted media controls (Figure S1). Percentages of plant wilting were 73.96 ± 5.91 and 67.71 ± 4.68 in water and media controls, respectively, whereas disease incidence in plants treated with supernatants of GLSH03, H8-1, and K203 was 40.63 ± 2.13, 43.75 ± 4.27, and 43.75 ± 2.28, respectively. The results of AUDPC tended to be similar to those of disease incidence (Figure S1). Supernatants of GLSH03, H8-1, K203, and streptomycin as a positive control showed significant differences from the controls. The AUDPC was 484.38 ± 43.89 in control (media) plants, while it was 353.13 ± 34.83, 287.33 ± 28.37, and 354.17 ± 28.76 in supernatants of GLSH03-, H8-1-, and K203-treated plants, respectively (Figure S1).

### 3.3. Bacterial Identification by 16S rRNA Gene Sequencing and Characterization

Strains GLSH03, H8-1, and K203 belong to the genus *Bacillus*, based on 16s rRNA sequence analysis (Figure S2). As a result of comparison with sequences of type strains, the GLSH03 (1457 bp) showed 99.86% similarity to *Bacillus velezensis* (CR502^T^, AY603658), while K203 (1435 bp) and H8-1 (1428 bp) exhibited 99.86% and 100% similarity to *B. siamensis* (KCTC13613^T^, AJVF01000043) and *B. aryabhattai* (B8W22^T^, EF114313), respectively (Figure S2). To confirm their characteristics and classification, biochemical tests were performed using API 50CH/B and API ZYM. It was found that 17 types (glycerol, L-arabinose, D-glucose, D-fructose, inositol, D-mannitol, etc.) could be used in all tested strains (Table S1). In contrast, d-arabinose, l-xylose, methyl-β-d-xylopyranoside, and methyl-α-d-mannopyranoside could not be used in any strain. Three strains—GLSH03, H8-1, and K203—used esterase (C4) and esterase lipase (C8); however, 12 enzymes, including lipase and leucine arylamidase, were not available (Table S2).

### 3.4. Bioactive Extracts Derived from Bacterial Supernatants

As a result of measuring the viability of *Cmm* treated with solvent or water extracts of bacterial supernatants, the water extracts of GLSH03, H8-1, and K203 showed inhibitory activity against *Cmm* viability. However, no activity was shown for any of the tested solvent extracts (data not shown). Regression curves of water extracts of GLSH03, H8-1, and K203 were y = 0.00000224x^2^ − 0.0005x + 0.8223, y = 0.0000018x^2^ − 0.0041x + 0.6891, and y = 0.00000336x^2^ − 0.0055x + 0.8281; the minimum inhibitory concentrations (MICs) of water extracts of GLSH03, H8-1, and K203 were 180.89 mg/mL, 157.24 mg/mL, and 138.51 mg/mL, respectively, while their lethal doses (LD_50_) were 124.86 mg/mL, 81.15 mg/mL, and 72.02 mg/mL, respectively (data not shown).

### 3.5. Biocontrol Activity in Plant Assays

**Figure 1:**
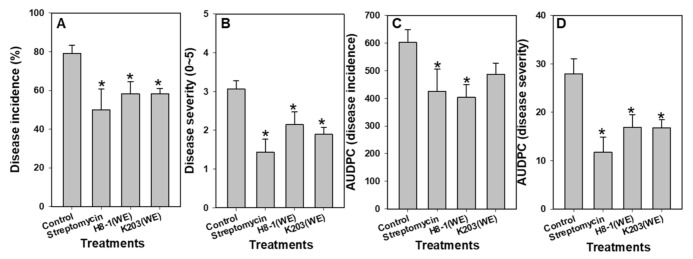
Disease incidence (**A**), severity (**B**), and area under the disease progress curve (AUDPC) (**C**,**D**) caused by *Clavibacter michiganensis* subsp. *michiganensis* (*Cmm*) in tomato plants. The mixtures of the water extracts and pathogen suspensions were treated in pots (final concentration: water extract, 1000 μL/g of soil, *Cmm*, 10^9^ cfu/g of soil). An asterisk on the bar indicates statistically significant difference compared to controls based on LSD (*p* < 0.05); error bars indicate standard errors (*n* = 6; 3 replicates of 8 plants per treatment). WE: water extract.

For determination of concentration for biocontrol assays in tomato plants, various concentrations (0, 10, 100, and 1000 μg/g) of three bacterial water extracts were tested. When the water extracts of H8-1 and K203 at 1000 μg/g were used, the disease incidence and severity were the most effectively suppressed, with control efficacies of 79.17% and 68.73% compared to the control, respectively (Figure S3). However, water extract of GLSH03 had suppressive activity on disease severity at only 10 μg/g (Figure S3). Disease incidence in tomato plants treated with water extracts of H8-1 and K203 was 58.33 ± 6.18 and 58.33 ± 2.63, respectively, and 79.17 ± 4.17 in control plants. Disease severity and the AUDPC of both also showed a significant difference in all treatments compared to the control, except for the AUDPC of disease incidence in the K203 treatment (Figure 1). As a result of the plant growth promotion test, there was a significant increase in K203-water-extract-treated plants compared to controls. In contrast, H8-1 was not different from controls. Shoot weights were 65.75 ± 4.34 g/plant in control plants, 68.83 ± 3.57 g/plant in H8-1-, and 78.27 ± 2.30 g/plant in K230-water-extract-treated plants, which was a 1.19-fold increase compared to the controls (Figure S4).

### 3.6. Colonization of Cmm in Tomato Plants

**Figure 2:**
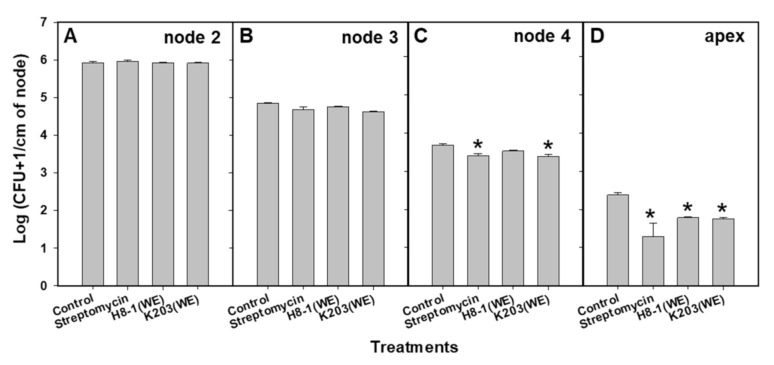
Colonization of *Cmm* in tomato plants at 20 days after inoculation. Stem segments (node 2 (**A**), node 3 (**B**), node 4 (**C**), and apex (**D**)) were homogenized and cultured on bacterial canker of tomato (BCT) media. An asterisk on the bar indicates significant statistical difference compared to controls, based on LSD (*p* < 0.05) (*n* = 10). WE: water extract.

Based on *Cmm* colony counting as intervals of time and location of nodes in tomato plants, treatment with water extracts of H8-1 and K203 suppressed colonization of *Cmm* in tomato plants at 20 days after inoculation (Figure 2). One day after inoculation, *Cmm* was not detected in any node of tomato plants; three days after inoculation, *Cmm* was detected in the first and second nodes of all treated plants (Figure S5). However, when tomato plants were treated with the water extract of H8-1 and streptomycin, the population of *Cmm* in the first and second nodes was significantly reduced compared to the controls (Figure S5). Seven days after inoculation, the *Cmm* population in tomato plants treated with the water extract of H8-1 and streptomycin was significantly (*p* < 0.05) reduced compared to that in the control plants (Figure S5). *Cmm* colonized in the apex of tomato plants at 15 days after inoculation; first, fourth, and apex nodes of tomato plants treated with water extract of H8-1 differed significantly from controls (Figure S5). Twenty days after inoculation, the *Cmm* population was significantly reduced in the fourth node of the K203-water-extract- and streptomycin-treated plants, and in the apex of the H8-1- (1.73 ± 0.02) and K203- (1.75 ± 0.04) water-extract-treated plants, compared to the controls (2.38 ± 0.06) (Figure 2).

### 3.7. Antioxidant Enzyme Activity in Tomato Plants

**Figure 3:**
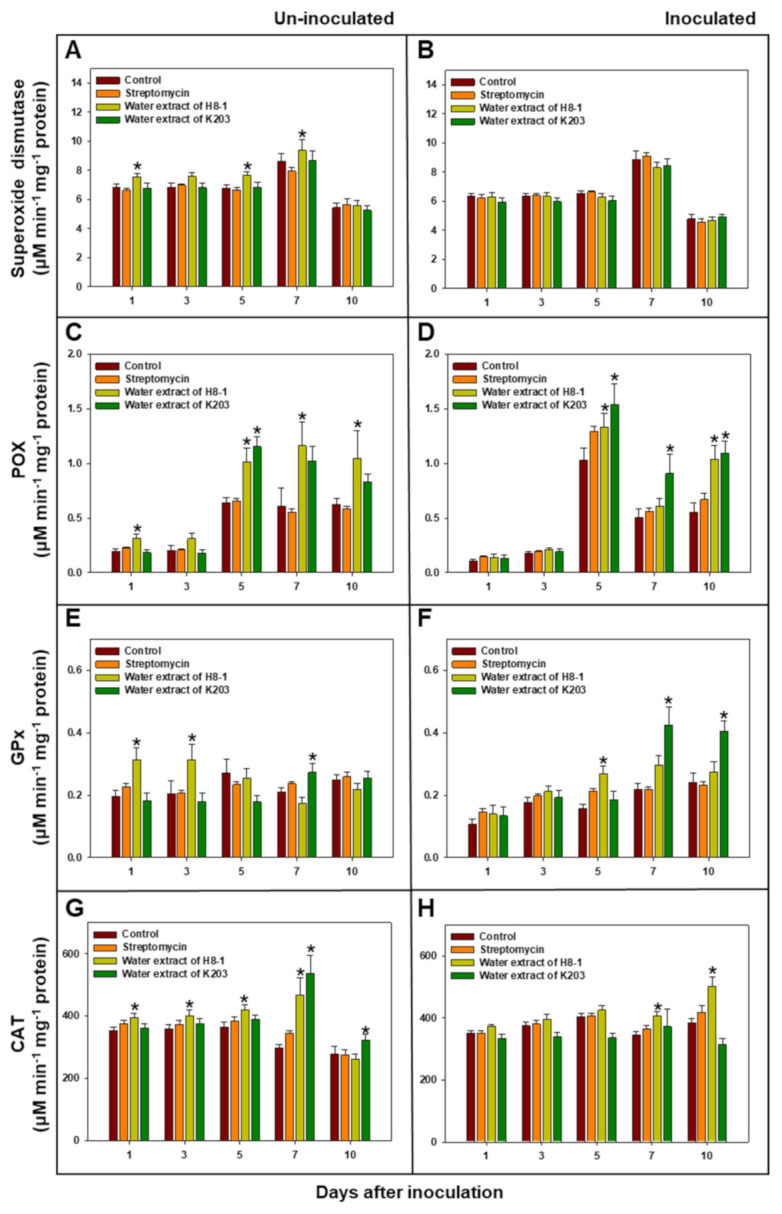
Activity of antioxidant enzymes such as superoxide dismutase (**A**,**B**), peroxidase (**C**,**D**), glutathione peroxidase (**E**,**F**), and catalase (**G**,**H**) by days after un- (**A**,**C**,**E**,**G**) or inoculation (**B**,**D**,**F**,**H**) of *Clavibacter michiganensis* subsp. *michiganensis* (*Cmm*). An asterisk on the bar indicates statistically significant difference compared to controls on each day after inoculation, based on LSD (*p* < 0.05); error bars indicate standard errors (*n* = 10).

Under non-inoculated conditions, water extracts of H8-1 and K203 tended to increase the levels of antioxidant enzymes—including peroxidase, glutathione peroxidase, and catalase—compared to the controls (Figure 3). Under *Cmm*-inoculated conditions, peroxidase activity in the H8-1-water-extract-treated plants at 5 and 10 DAI was 129.0% and 188.4% higher than in control plants; the activity in the K203-water-extract-treated plants at 5, 7, and 10 DAI increased by 148.8, 180.5, and 197.6%, respectively, compared to control plants. Glutathione peroxidase activity at 5 DAI in H8-1-treated plants, and 7 and 10 DAI in K203-treated plants, was significantly (*p* < 0.05) increased compared to control plants. Catalase activity increased only in the water extract of the H8-1-treated plants at 7 and 10 DAI compared to the control plants. However, superoxide dismutase activity did not show any difference between treatments in *Cmm*-inoculated plants (Figure 3).

### 3.8. Cmm Pathogenicity-Related Gene Expression

**Figure 4:**
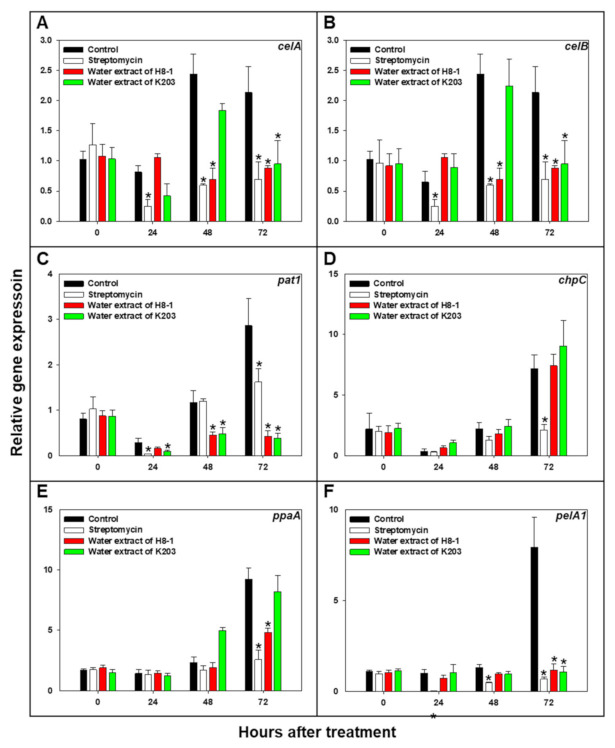
Relative transcript levels of *celA* (**A**), *celB* (**B**), *pat1*(**C**), *chpC* (**D**), *ppaA* (**E**), and *pelA1* (**F**) were determined by quantitative real-time polymerase chain reaction at various hours after treatment. Relative gene expression of *Clavibacter michiganensis* subsp. *michiganensis* (*Cmm*) grown in M9 medium amended with carboxymethyl cellulose (CMC) was normalized with *gyrA.* An asterisk on the bar indicates statistically significant difference compared to controls at each hour after treatment, based on LSD (*p* < 0.05); error bars indicate standard errors (*n* = 8).

The effect of the selected water extracts on the expression of the *celA*, *celB*, *pat1*, *chpC*, *ppaA*, and *pelA1* genes of *Cmm* was evaluated by qRT-PCR (Figure 4). When *Cmm* was treated with water extracts of H8-1 and K203, the relative gene expressions of *celA*, *celB*, *pat1*, and *pelA1* were significantly (*p* < 0.05) reduced compared to the controls at 72 h after treatment (HAT). Relative expression of *celA* was suppressed by 0.41-fold in H8-1-water-extract-treated *Cmm* and 0.45-fold in K203-water-extract-treated *Cmm* at 72 HAT; *celB* was less expressed by 0.01-fold in H8-1-water-extract-treated *Cmm* and 0.02-fold in K203-water-extract-treated *Cmm* at 72 HAT. In the case of *pat1* and *pelA1* gene expression at 72 HAT, when treated with water extract of H8-1, their expression was reduced by 0.15- and 0.14-fold, respectively; when treated with water extract of K203, both were decreased by 0.13-fold. The *ppaA* gene expression of *Cmm* was significantly (*p* < 0.05) decreased in treatments of H8-1 water extract and streptomycin at 72 HAT. Streptomycin, as a positive control, reduced the relative expression of the *celA*, *celB*, *pat1*, *chpC*, *ppaA*, and *pelA* genes of *Cmm* at 72 HAT (Figure 4).

### 3.9. Relative Expression of Plant Genes, including ACO, PI2, and PR1a

**Figure 5:**
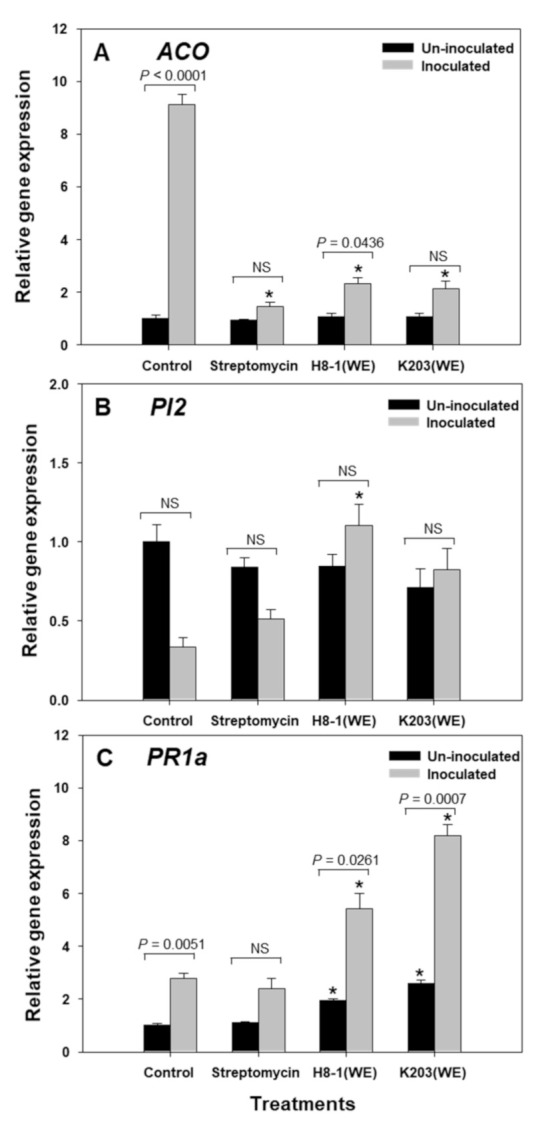
Relative transcript levels of *ACO* (**A**)*, PI2* (**B**), and *PR1a* (**C**) were determined by quantitative real-time polymerase chain reaction at 72 h after inoculation (HAI). Relative expression levels of *ACO, PI2,* and *PR1a* in *Clavibacter michiganensis* subsp. *michiganensis* (*Cmm*)-infected plants were normalized with *GAPDH*. An asterisk on the bar indicates statistically significant difference between treatments in inoculated or non-inoculated plants, based on LSD (*p* < 0.05); *p*-values on the bars represent comparisons between non-inoculated and inoculated plants; error bars indicate standard errors (*n* = 8). NS: not significant.

Under non-inoculated conditions, the relative expression of *ACO* and *PI2* did not differ between treatments; however, under *Cmm*-inoculated conditions, water extracts of H8-1 and K203 decreased the gene expression of *ACO* compared to the control at 72 HAI (Figure 5). *PI2* gene expression was increased significantly by treatment with H8-1 water extract at 72 HAI in *Cmm*-inoculated plants. In H8-1- and K203-water-extract-treated plants, *PR1a* was expressed more highly than in control plants at 72 HAI, in both non-inoculated and inoculated conditions (Figure 5). As a comparison of non-inoculation and *Cmm* inoculation, when plants were inoculated with *Cmm*, tomato *ACO* and *PR1a* genes’ expression was increased, while *PI2* expression did not show any differences. As a result of the plant gene expression, water extracts of H8-1 and K203 reduced *ACO* expression, and further enhanced *PR1a* gene expression compared to controls under *Cmm* inoculation.

### 3.10. Soil Microbial Activity

**Figure 6:**
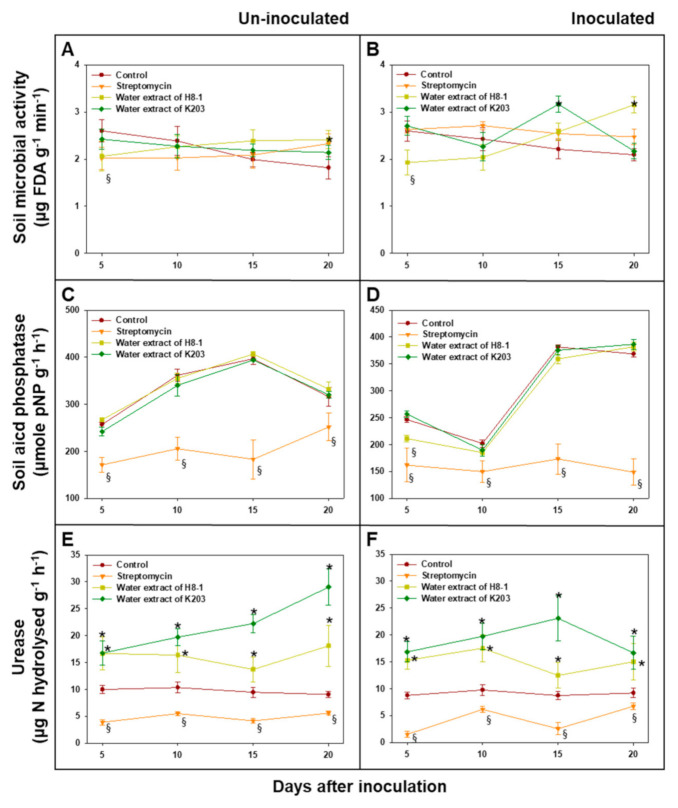
Total microbial activity (**A**,**B**) and soil enzyme activity such as acid phosphatase (**C**,**D**), and urease (**E**,**F**) by days after un- (**A**,**C**,**E**) or inoculation (**B**,**D**,**F**) with *Clavibacter michiganensis* subsp. *michiganensis* (*Cmm*) and extract treatment in tomato plants. Asterisks and § on the bar indicate statistically significant differences as higher and less than controls, based on LSD (*p* < 0.05), respectively; error bars indicate standard errors (*n* = 10).

**Figure 7:**
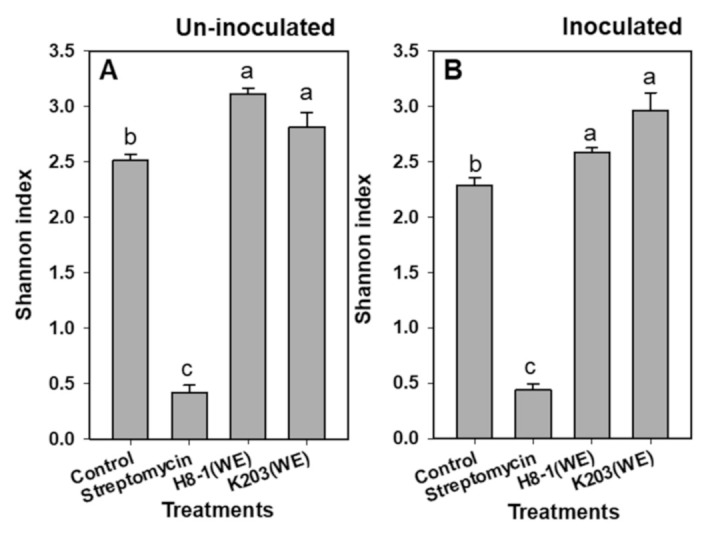
Shannon’s diversity index based on EcoPlates of rhizosphere soils taken from *Clavibacter michiganensis* subsp. *michiganensis* (*Cmm*) un- (**A**) or inoculated (**B**) tomato plants. The final values of each well at 72 h, where H = –∑(Pi × lnPi), and Pi is the proportional optical density value of each well. The small letters (a–c) on the bar indicate statistically significant differences based on LSD (*p* < 0.05); error bars indicate standard errors (*n* = 10).

Total microbial activity assayed by fluorescein diacetate hydrolysis in tomato-grown soils treated with the K203 water extract showed higher levels than the controls 15 days after inoculation (DAI), whereas, in the case of H8-1, soil microbial activity was increased regardless of *Cmm* inoculation compared to that of the controls 20 DAI (Figure 6). Under *Cmm*-inoculated and non-inoculated conditions, soil acid phosphatase activity was not significantly different between water extracts and controls, except at 5 DAI in soils inoculated with *Cmm*. Urease activity in soils treated with water extracts of H8-1 and K203 was higher than that in the controls during the test period, regardless of *Cmm* inoculation (Figure 6). In contrast, when soils were treated with streptomycin, the soil acid phosphatase and urease activities were lower than those of the controls. As a result of community analysis using Biolog EcoPlate^TM^ based on the influence of microbial carbon substrate use, the Shannon index for species diversity significantly increased when water extracts of H8-1 and K203 were used for treatment, regardless of *Cmm* inoculation; however, streptomycin-treated soils had reduced community diversity compared to the controls (Figure 7).

## 4. Discussion

In this study, we found that bioactive water extracts derived from *Bacillus* strains H8-1 and K203 suppressed tomato wilt caused by *Cmm*. The two bacterial supernatants inhibited the *Cmm*’s viability and suppressed the secretion of cellulase, which is a pathogenic factor of *Cmm* [34]. When *Cmm* was treated with the extracts, the *celA*, *celB* (cellulase), *pat1*, and *pelA1* (pectate lyase) of *Cmm* showed suppressed expression. In the plant assay, water extracts of H8-1 and K203 significantly reduced disease incidence and severity, together with reducing ethylene-related gene (*ACO*) expression and increasing *PR-1a* gene expression. Moreover, the extracts tended to limit the colonization and development of *Cmm* in the upper part of the tomatoes’ interior. Additionally, water extracts could affect the soil microbial activity and diversity.

The water extracts of H8-1 and K203 significantly suppressed tomato wilt caused by *Cmm* in the pot assay. This effect could be explained by the following factors: For successful infection of host plants, *Cmm* secretes various enzymes, including cellulase and pectate lyase [11]. *Cmm* has two major cellulase genes—*celA* and *celB*—and the pathogen uses the secretion enzymes to enter the host plant via maceration [3]. Previous studies have shown that *celA* is a major virulence factor that causes wilt in tomatoes, playing a pivotal role in virulence function and promoting host infection by *Cmm* [34,46]. Supernatants containing water extracts of H8-1 and K203 directly reduced *Cmm* viability and cellulase activity. Moreover, the water extracts of H8-1 and K203 decreased the expression of *Cmm* genes, such as *celA* (cellulase), which is pivotal for entering tomato plants. Therefore, the water extracts of H8-1 and K203 could decrease the possibility of infection by attenuating virulence functions such as cellulase activity or secretion. In addition to *celA* and *celB,* expression of *pat1* and *pelA1* genes was also affected by the water extracts of H8-1 and K203. Like *celA*, *pelA1* also participates in host cell degradation during host plant invasion [11]. Chalupowicz et al. [39] found that the transcription of chromosomal genes involved in cell wall degradation, such as *pelA1* and *celB*, was induced during early infection, and proteases encoded by *pat1*, *chpC*, *and ppaA* are involved in host colonization, acquisition of the pathogen’s nutrient sources, and attenuation of host defenses [10,11]. According to the results of Stork et al. [47] and Chalupowicz et al. [6], mutation of *chpC*—which participates in both virulence and colonization—dramatically decreases disease, limiting it to only weak symptoms. In addition to the effects on *Cmm* virulence gene expression, when both water extracts were used, *Cmm* was restricted in colonization and development in the tomatoes’ interior, from the root to the upper node of the stem. Therefore, the water extracts of H8-1 and K203 might affect *Cmm* virulence gene expression related to cell degradation enzymes, such as cellulase and protease, restricting the colonization and development of *Cmm* in the xylem of tomato plants, as well as infection.

The water extracts of H8-1 and K203 affected the plant-defense-related responses. Antioxidant enzyme levels were increased regardless of *Cmm* inoculation in the water-extract-treated-plants; similarly, when DL-β-aminobutyric acid or acibenzolar-S-methyl were added, the levels of reactive oxygen species (ROS)-scavenging antioxidant systems—including peroxidase, phenylalanine ammonia-lyase, and glutathione peroxidase—were increased [33,48,49]. Antioxidant enzymes also play important roles in the reinforcement of plant cell walls by increasing phenols and lignin; consequently, they can act as a physical barrier against penetration by cell wall degradation enzymes of *Cmm* [50]; therefore, the treatments with water extracts can help to enhance plant defense against *Cmm* infection by increasing antioxidant enzyme activities. Meanwhile, water extracts can also alter the expression of plant genes, such as *PR1a*, *PI2*, and *ACO*. Plant *PR1a* has been used as a marker gene for inducing salicylic acid (SA)-dependent resistance [51,52]. *PR1a* gene expression was significantly increased in the water-extract-treated plants; therefore, the extracts might enhance plant defense responses against *Cmm* via salicylic-acid-dependent pathways and suppress tomato wilt. The *PI2* gene, which encodes a proteolytic enzyme inhibitor used as a jasmonic-acid-dependent marker gene, can be triggered by wounding [40]. In this study, the *PI2* gene dramatically increased in plants treated with H8-1-water extract at 72 h after inoculation; therefore, H8-1-water extract can be involved in a jasmonic-acid-dependent defense pathway. During *Cmm* infection, 1-aminocyclopropane-1-carboxylic acid (ACC)-oxidase as an ethylene-synthesizing enzyme was induced, and *Cmm*-triggered ethylene synthesis in host plants could be be one of the important factors in disease development [12,53]. As a result of *ACO* encoding 1-aminocyclopropane-1-carboxylic acid (ACC) oxidase expression, *ACO* gene expression significantly decreased in water-extract-treated plants, corresponding to disease suppression.

Additionally, the two extracts tended to increase the soil microbial activity, urease activity, and microbial diversity. This suggests that the water extract can affect soil microbial diversity, which can promote soil health correlated with microbial activity, without negative influence. The increase in shoot weight in water-extract-treated plants may also be related to the use of microbial carbon sources and soil urease activity [54]. Therefore, the extracts might affect soil microbial environments, and could indirectly affect soil-borne pathogens, such as *Cmm*. Here, the water extract of *Bacillus* strains H8-1 and K203 suppressed pathogenicity factors such as cell degradation enzymes of *Cmm*, which are necessary for infection, and diminished colonization and development into the xylem of tomato plants. Moreover, the extracts may affect the plants’ ROS-scavenging systems involved in the defense response, and induce *PR1a* gene expression in a salicylic-acid-dependent manner, as well as soil microbial activities. Taken together, the water extracts of H8-1 and K203 could directly inhibit *Cmm* and indirectly induce plant defense and affect soil microbial activities; therefore, the water extracts could be used as biocontrol agents for controlling tomato bacterial wilt caused by *Cmm*.

## Figures and Tables

**Table 1:** Primers of pathogenicity-related genes of *Clavibacter michiganensis* subsp. *michiganensis* (*Cmm*).

*Cmm* Genes	Sequence (5′ → 3′)	Expressed Gene	References
Gyrase	Forward: GTGGTCGGCGAGGTC Reverse: GCGCGAGCGGGTAG	*gyrA*	[39]
Cellulase A	Forward: GGTTCTCCGCATCAAACTATCC Reverse: TGCTTGTCGCTCGTCGTC	*celA*	
Cellulase B	Forward: GGAGACCACCAGCGACAAG Reverse: TGAACGACCAGAACGACGAG	*celB*	
Serine protease	Forward: GCTGATTCGCGAGAGGATC Reverse: GTTCTCGGTTGCTGTGTCGC	*pat-1*	
*Chp* family protease	Forward: GACTGCTAATCACTGTGTTG Reverse: CAATAAACCGTTCCGATGG	*chpC*	
Chymotrypsin-related serine protease	Forward: AATCGGGCTGGTTCTGGTTT Reverse: AGATTCTGCGGCATCTGCAT	*ppaA*	
Pectinase	Forward: GTGCGTTCCTGCGGTAAC Reverse: GCGGATGGTGATGTGGTC	*pelA1*	

**Table 2:** Primers of defense-related genes of tomato.

Tomato Plant Genes	Sequence (5′ → 3′)	Expressed Gene	Reference
Pathogenesis-related protein	Forward: GTGGGATCGGATTGATATCCT Reverse: CCTAAGCCACGATACCATGAA	*PR1a*	[40]
Proteinase inhibitor	Forward: AATTATCCATCATGGCTGTTCAC Reverse: CCTTTTTGGATCAGATTCTCCTT	*PI2*	
1-Aminocyclopropane-1-carboxylix acid oxidase	Forward: AAGATGGCACTAGGATGTCAATAG Reverse: TCCTCTTCTGTCTTCTCAATCAAC	*ACO*	
Glyceraldehyde 3-phosphate dehydrogenase	Forward: CTGGTGCTGACTTCGTTGTTG Reverse: GCTCTGGCTTGTATTCATTCTCG	*GAPDH*	

**Table 3:** Relative cell viability and cellulase activity of *Clavibacter michiganensis* subsp. *michiganensis*.

Treatments	Inhibition of *Cmm* Viability ^a^	Cellulase Activity	Treatments	Inhibition of *Cmm* Viability	Cellulase Activity	Treatments	Inhibition of *Cmm* Viability	Cellulase Activity	Treatments	Inhibition of *Cmm* Viability	Cellulase Activity
GLSH03	98.32 ± 0.22 *	-	TS6-3	57.15 ± 1.42 *	+	GLS09	22.82 ± 2.33 *	+	JC72	5.21 ± 0.21	+
HN12	98.27 ± 0.12 *	-	JC35	53.70 ± 1.62 *	+	GTH05	22.60 ± 2.55 *	-	GLCH05	4.85 ± 0.07	+
H8-1	98.06 ± 0.03 *	-	TS5-2	51.78 ± 2.15 *	+	JTL04	22.10 ± 3.22 *	-	JC39	1.35 ± 0.30	-
JC34	97.91 ± 0.11 *	+	MB7-3	48.88 ± 2.40 *	-	JTL08	21.54 ± 3.27 *	-	JC18	−1.66 ± 6.73	+
K203	97.71 ± 0.26 *	-	JC46	45.07 ± 0.62 *	+	JC54	20.82 ± 1.91 *	+	H19-1	−3.15 ± 1.49	+
H2-7	97.51 ± 0.07 *	-	GLSH04	41.65 ± 1.99 *	-	GCH09	20.61 ± 2.20 *	+	JC55	−3.82 ± 3.06	+
5GH 41-08	97.39 ± 0.02 *	+	GLSH01	41.60 ± 0.68 *	-	HN02	18.61 ± 2.86 *	-	JC12	−3.84 ± 0.02	-
H24-9	96.82 ± 0.15 *	+	H18-10	41.30 ± 1.81 *	-	HN09	18.29 ± 3.71 *	-	HN20	−4.48 ± 3.90	+
TS3-1	95.76 ± 0.26 *	-	GLCH06	40.31 ± 0.75 *	-	HN11	17.73 ± 3.58 *	-	JC26	−12.20 ± 4.67	-
HN05	95.31 ± 0.06 *	-	JC41	37.56 ± 1.21 *	-	MB7-1	16.88 ± 5.41 *	-	HN29	−12.50 ± 6.38	-
GTH01	92.78 ± 1.51 *	+	GLSH06	36.89 ± 2.63 *	-	GLSH10	16.83 ± 1.02 *	+	5GH31-15	−14.71 ± 5.85	+
JC33	90.61 ± 0.81 *	+	TS7-2	34.92 ± 1.19 *	+	JC16	16.73 ± 3.39 *	-	H12-10	−15.01 ± 5.40	+
H8-5	76.80 ± 1.92 *	+	H6-4	34.65 ± 3.70 *	-	GLC09	16.66 ± 2.18 *	-	H1-2	−17.21 ± 3.61	+
H33-8	74.94 ± 1.91 *	+	GCH06	34.58 ± 1.57 *	+	GCH05	16.24 ± 0.88 *	+	JTL02	−17.66 ± 0.42	-
TS6-1	71.41 ± 0.76 *	+	JC53	33.38 ± 3.67 *	-	H6-7	15.86 ± 5.30	-	H1-1	−23.42 ± 1.84	-
K204	69.73 ± 0.19 *	+	GLSH08	32.97 ± 4.06 *	-	GLCH04	14.14 ± 3.99	+	JTL05	−23.48 ± 5.89	-
5GH 41-07	69.16 ± 0.47 *	+	H23-8	32.76 ± 3.50 *	+	H20-5	13.95 ± 3.62	+	JTL03	−26.26 ± 3.37	-
H30-3	68.37 ± 1.49 *	+	HN10	29.51 ± 0.34 *	-	HN24	12.37 ± 5.06	+	H1-8	−29.74 ± 2.09	+
MB5-1	67.85 ± 0.76 *	+	JC08	28.35 ± 3.20 *	-	GLSH09	12.29 ± 4.50	-	JC27	−31.48 ± 4.00	+
GLC02	63.71 ± 1.89 *	+	GLCH09	26.76 ± 5.73 *	+	HN22	10.03 ± 4.10	-	JC28	−40.70 ± 3.22	+
H30-6	60.23 ± 1.82 *	+	HN03	26.18 ± 3.15 *	+	GLCH03	10.01 ± 3.98	+	JTL06	−55.43 ± 6.10	-
MB7-5	59.51 ± 0.66 *	+	HN08	26.18 ± 1.49 *	+	JC59	9.82 ± 1.73	+	JTR09	−55.44 ± 1.89	+
K185	58.63 ± 2.05 *	+	HN25	24.06 ± 4.56 *	+	JTR01	9.48 ± 4.28	-	GLSH11	−72.94 ± 5.66	-
H15-2	58.19 ± 2.66 *	+	H5-9	23.58 ± 3.64 *	-	GC03	6.58 ± 1.68	+			

^a^: Relative reduction in cell viability (%) of *C. michiganensis* subsp. *michiganensis* (*Cmm*) treated with tenfold-diluted supernatant; +: production of cellulase was higher than in non-treated *Cmm*; -: production of cellulase was lower than that in non-treated *Cmm*. An asterisk means statistical difference compared to controls, based on LSD (*p* < 0.05).

## Data Availability

Not applicable.

## Supplementary Materials

The following supporting information can be downloaded at: https://www.mdpi.com/article/10.3390/microorganisms10020403/s1, Figure S1: Disease suppression by bacterial supernatants in tomato plant, Figure S2: Phylogenetic analysis of 16S rRNA sequences of strains GLSH03, H8-1, and K203, Figure S3: Selection of water extract-concentration for controlling the disease caused by *Clavibacter michiganensis* subsp. *michiganensis* (*Cmm*), Figure S4: Plant growth promotion by the water extract, Figure S5: Colonization of *Clavibacter michiganensis* subsp. *michiganensis* (*Cmm*) in tomato plants, Table S1: API 50CH test of strains K203, H8-1 and GLSG03, Table S2: API ZYM test of strains K203, H8-1 and GLSH03.
