# Supplementary material for: Suppressive Effect of Bioactive Extracts of Bacillus sp. H8-1 and Bacillus sp. K203 on Tomato Wilt Caused by Clavibacter michiganensis subsp. michiganensis
Source: Microorganisms. 2022 Feb 9;10(2):403. doi: 10.3390/microorganisms10020403 (PMC8880269; doi:10.3390/microorganisms10020403)
Supplement: Supplementary file 1 [file microorganisms-10-00403-s001.zip › microorganisms-1546066-SI.pdf]

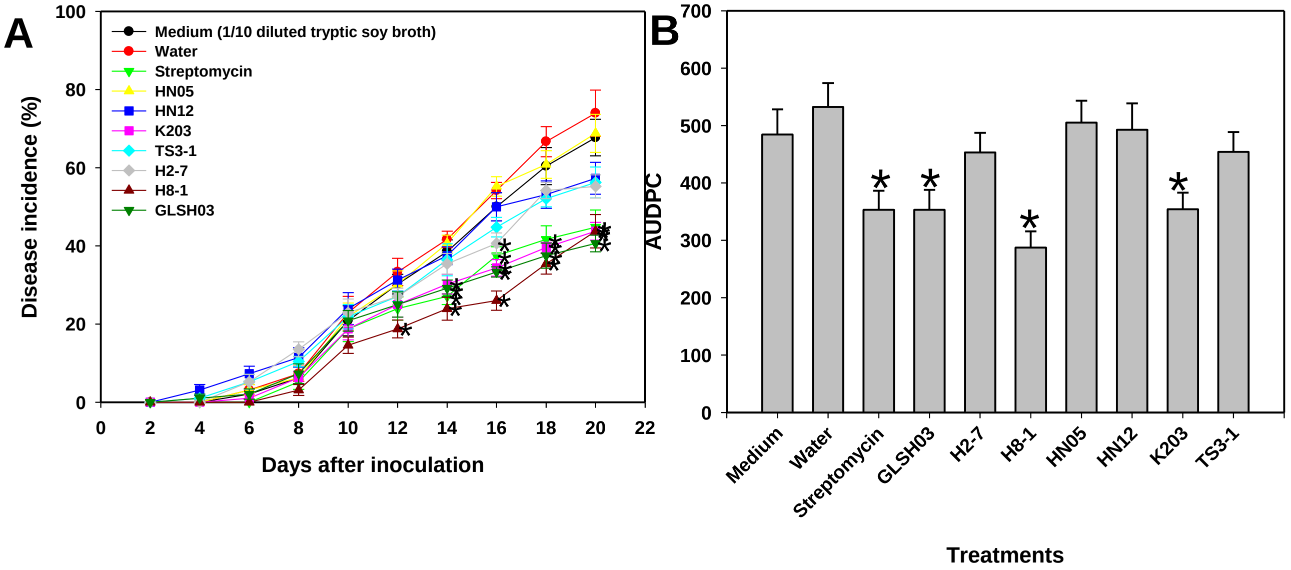

Figure S1. Disease incidence (A) and AUDPC (B) caused by *Clavibacter michiganensis* subsp. *michiganensis (Cmm)* in tomato plant. The bacterial supernatants and *Cmm* suspension were treated into pots. An asterisk on the bar mean statistical difference compared to medium by LSD (*P* < 0.05) and error bars indicate standard errors (three replicates of 8 plants per treatment).

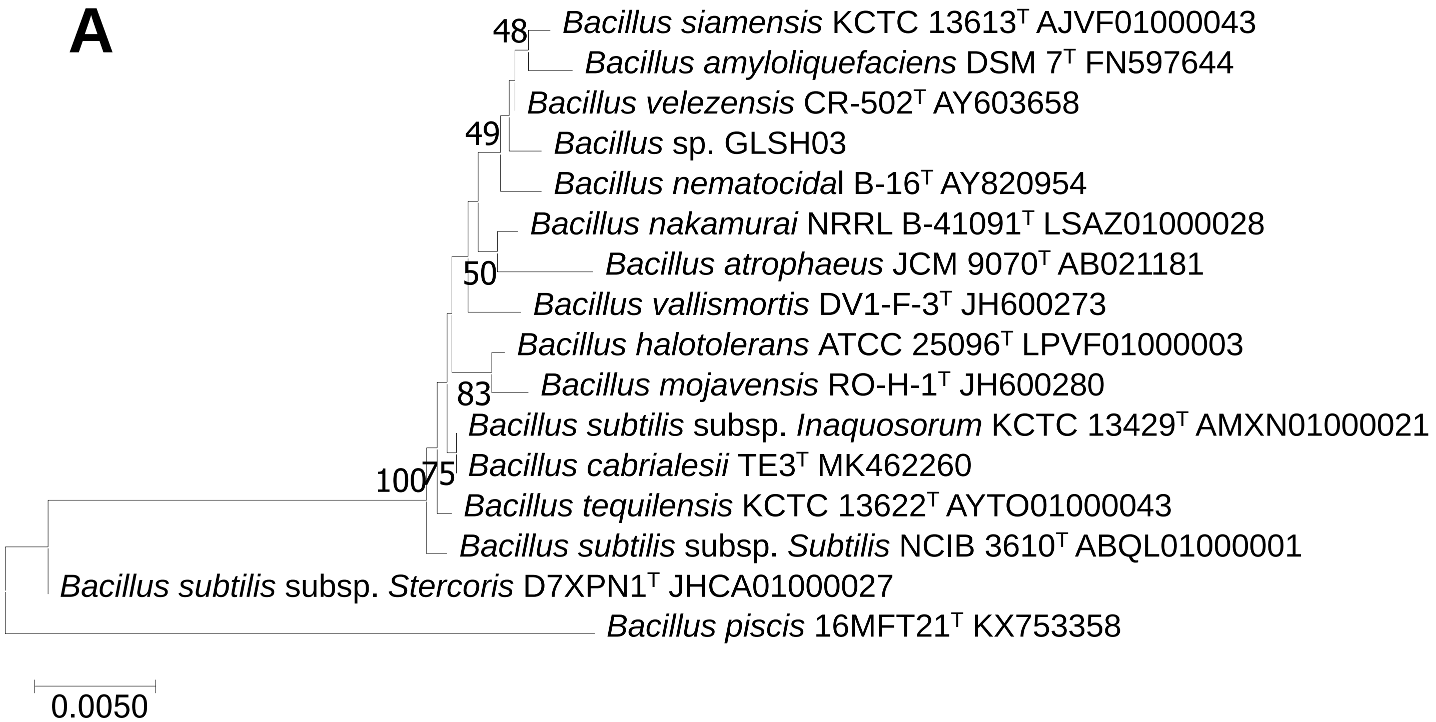
A
48
49
50
83
100
75
Bacillus siamensis KCTC 13613T AJVF01000043
Bacillus amyloliquefaciens DSM 7T FN597644
Bacillus velezensis CR-502T AY603658
Bacillus sp. GLSH03
Bacillus nematocidal B-16T AY820954
Bacillus nakamurai NRRL B-41091T LSAZ01000028
Bacillus atrophaeus JCM 9070T AB021181
Bacillus vallismortis DV1-F-3T JH600273
Bacillus halotolerans ATCC 25096T LPVF01000003
Bacillus mojavensis RO-H-1T JH600280
Bacillus subtilis subsp. Inaquosorum KCTC 13429T AMXN01000021
Bacillus cabrialesii TE3T MK462260
Bacillus tequilensis KCTC 13622T AYTO01000043
Bacillus subtilis subsp. Subtilis NCIB 3610T ABQL01000001
Bacillus subtilis subsp. Stercoris D7XPN1T JHCA01000027
Bacillus piscis 16MFT21T KX753358
0.0050

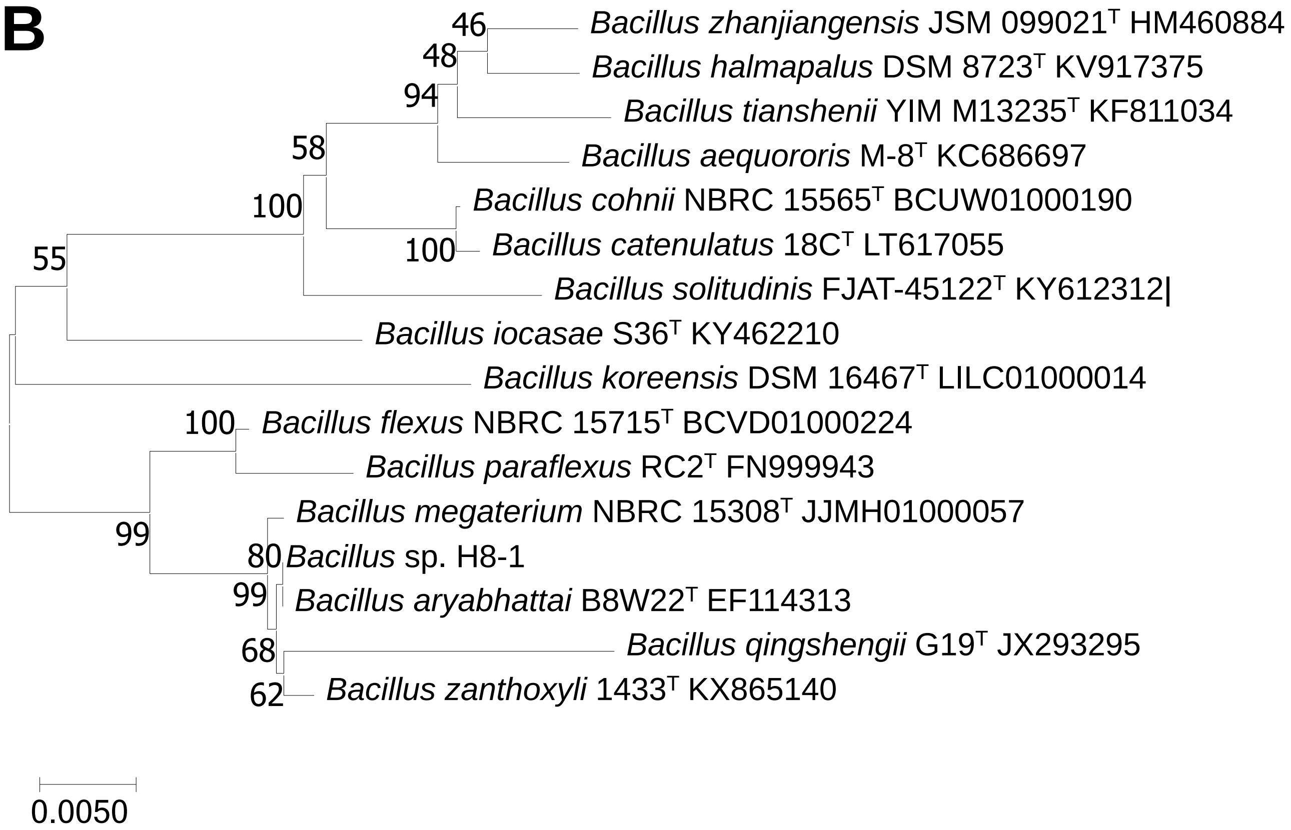
B
46
48
94
58
100
100
55
100
99
80
99
68
62
Bacillus zhanjiangensis JSM 099021T HM460884
Bacillus halmapalus DSM 8723T KV917375
Bacillus tianshenii YIM M13235T KF811034
Bacillus aequororis M-8T KC686697
Bacillus cohnii NBRC 15565T BCUW01000190
Bacillus catenulatus 18CT LT617055
Bacillus solitudinis FJAT-45122T KY612312|
Bacillus iocasae S36T KY462210
Bacillus koreensis DSM 16467T LILC01000014
Bacillus flexus NBRC 15715T BCVD01000224
Bacillus paraflexus RC2T FN999943
Bacillus megaterium NBRC 15308T JJMH01000057
Bacillus sp. H8-1
Bacillus aryabhattai B8W22T EF114313
Bacillus qingshengii G19T JX293295
Bacillus zanthoxyli 1433T KX865140
0.0050

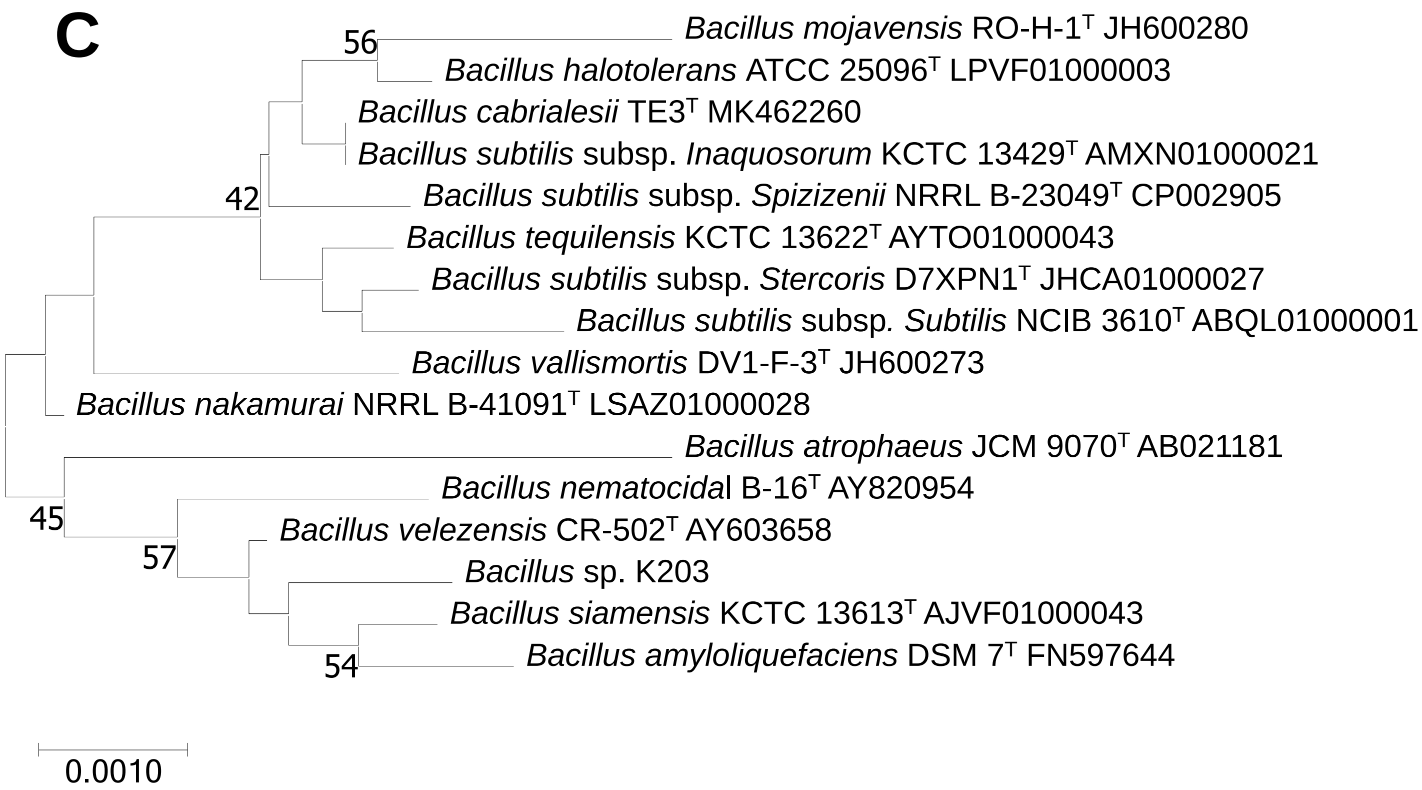

Figure S2. Phylogenetic trees composed by neighbor-joining method based on 16S rRNA sequences of strains GLSH03 (A), H8-1 (B), K203(C) Bootstrap values based on 1,000 replications are shown at the branch points.

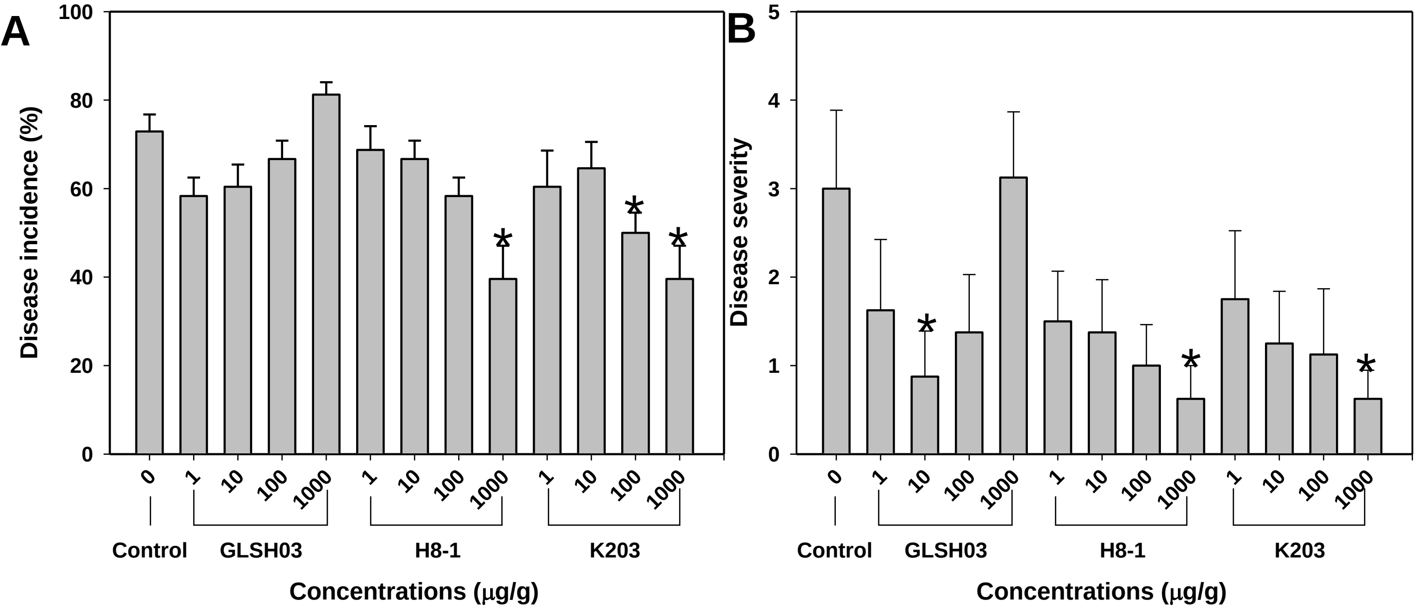

Figure S3. Disease incidence (A) and severity (B) caused by *Clavibacter michiganensis* subsp. *michiganensis* (*Cmm*) in tomato plant. The mixures of filtered water extracts and *Cmm* suspension were treated into pots (final concentration : Water extract, 1, 10, 100 and 1,000 ul/g of soil, *Cmm*, 10^9 cfu/g of soil). An asterisk on the bar mean statistical difference compared to control by LSD ($P < 0.05$) and error bars indicate standard errors (three replicates of 8 plants per treatment).

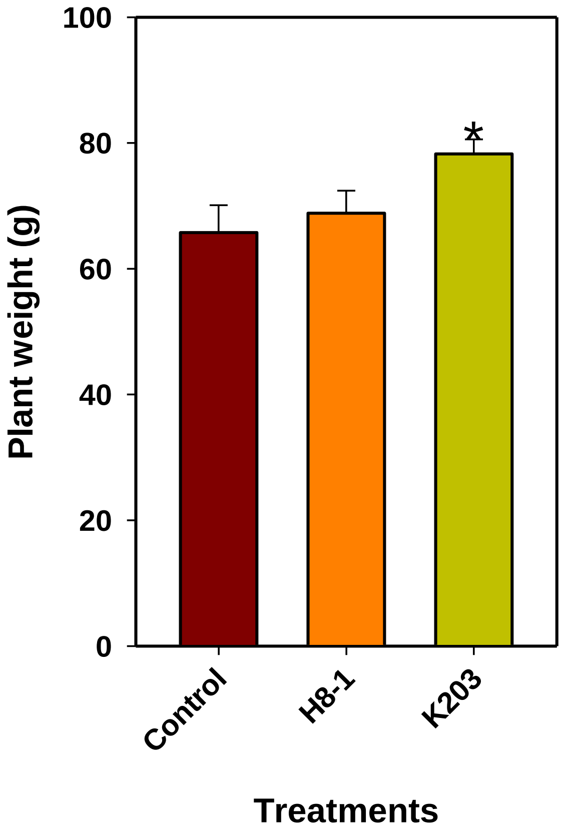

Figure S4. Shoot weight (g plant^{-1}) after treatment of the bacterial water extract. An asterisk on the bar mean statistical difference by LSD (P < 0.05) and error bars indicate standard errors (three replicates of 8 plants per treatment).

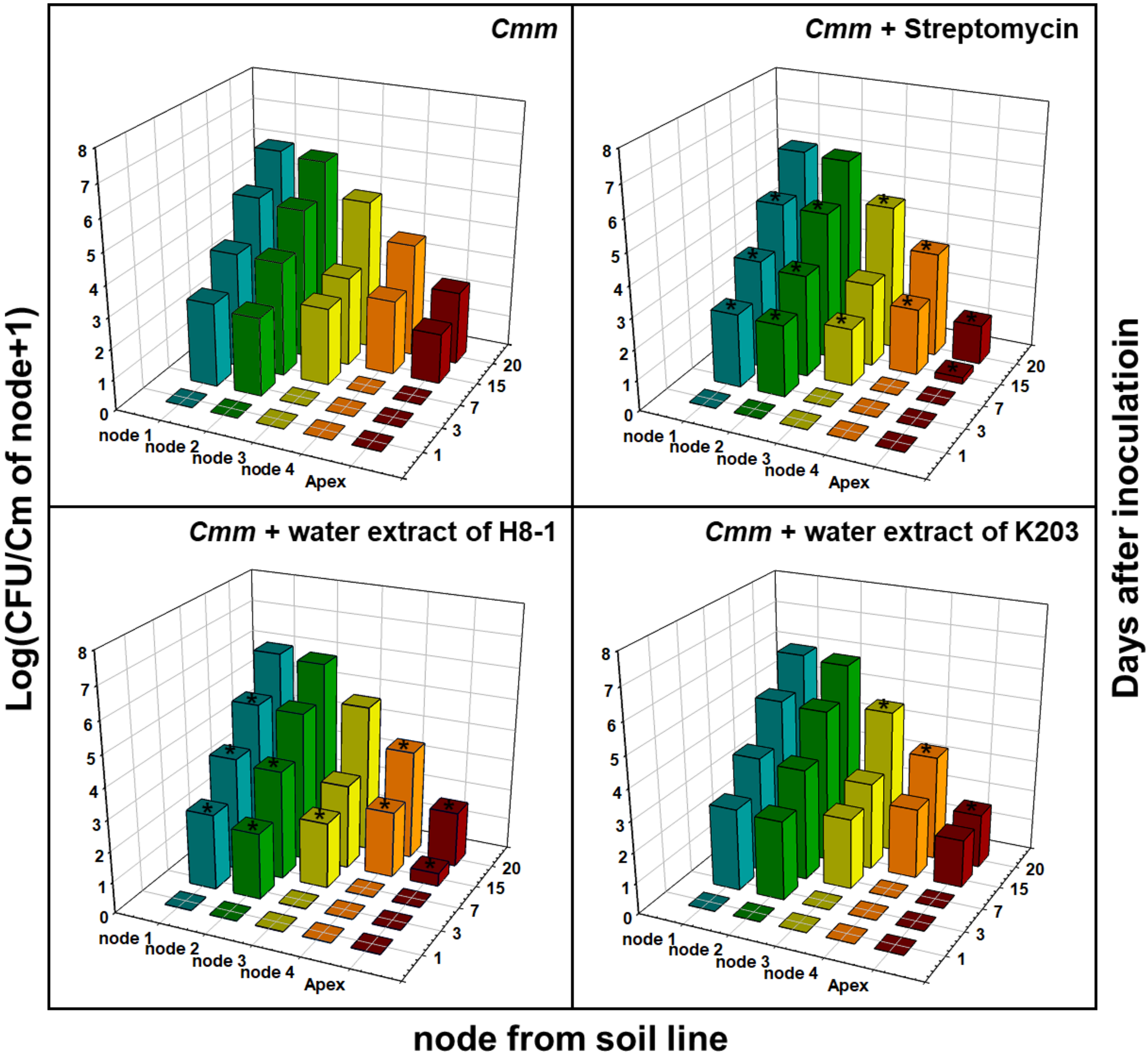

Figure S5. Colonization of *Clavibacter michiganensis* subsp. *michiganensis* (*Cmm*) in tomato plants. Stem segments were homogenized, and cultured on bacterial canker of tomato (BCT) mediums. An asterisk on the bar means significant statistical difference compared to control by LSD ($P < 0.05$) (n=10).

Table S1. API 50CH tests of strains K203, H8-1 and GLSH03 at 24 hours

Conponent	K203	H8-1	GLSH03
control			
glycerol	±	±	±
erythritol	±	-	-
D-arabinose	-	-	-
L-arabinose	±	±	±
D-ribose	-	±	±
D-xylose	±	-	±
L-xylose	-	-	-
D-adonitol	±	-	-
methyl-BD-xylopyranosicle	-	-	-
D-galactose	-	±	-
D-glucose	+	+	+
D-fructose	+	+	+
D-mannose	+	-	+
L-sorbose	-	-	±
rhamnose	-	-	±
dulcitol	-	±	±
inositol	±	±	+
mannitol	±	+	+
sorbitol	±	-	+
a-methyl-D-mannoside	-	-	-
a-methyl-D-glucoside	±	-	+
N-acethyl-glucosamine	±	±	+
amygdalin	±	+	+
arbutin	±	±	+
esculin	+	±	+
salicin	±	+	+
cellobiose	+	±	+
maltose	±	±	+
lactose	±	+	±
melibiose	-	±	+
sucrose	+	+	+
trehalose	±	+	-
inulin	±	+	-
melezitose	±	-	+
raffinose	±	+	+
starch	±	+	+
gltcogen	±	+	±
xylitol	-	+	+
gentiobiose	±	-	±
D-turanose	-	+	±
D-lyxose	-	±	±
D-tagatose	-	-	±
D-fucose	-	-	±
L-fucose	-	-	±
D-arabitol	-	-	±
L-arabitol	-	-	±
gluconate	-	-	±
2-keto-gluconate	-	-	±
5-keto-gluconate	-	-	±

+, Positive; , ±, intermediate; -, negativereactions

Table S2. API ZYM tests of strains K203, H8-1 and GLSH03 at 4H

Enzyme	Substrate	PH	K203	H8-1	GLSH03
Alkaline phosphatase	2-naphthyl phosphate	8.5	-	-	+
Esterase (C4)	2-naphthyl butyrate	6.5	+	+	+
Esterase Lipase (C8)	2-naphthyl caprylate	7.5	+	+	+
Lipase (C14)	2-naphthyl myristate	7.5	-	-	-
Leucine arylamidase	L-leucyl-2-naphthylamide	7.5	-	-	-
Valine arylamidase	L-valyl-2-naphthylamide	7.5	-	-	-
Crystine arylamidase	L-cystyl-2-naphthylamide	7.5	-	-	-
Trypsin	N-benzoyl-DL-arginine-2-naphthylamide	8.5	-	-	-
α–chymotrypsin	N-glutaryl-phenylanine-2-naphthylamide	7.5	-	-	-
Acid phospatase	2-naphtyl phosphate	5.4	-	+	-
Naphtol-AS-BI-phosphohydrolase	Naphthol-AS-BI-phosphate	5.4	-	+	+
α – galactosidase	6-Br-2-naphthyl-αD-galactopyranoside	5.4	-	+	-
β - galactosidase	2-naphthyl-βD-galactopyranoside	5.4	-	+	-
β - glucuronidase	Naphthol-AS-BI-βD-glucuronide	5.4	-	-	-
α – glucosidase	2-naphthyl-αD-glucopyranoside	5.4	-	-	-
β - glucosidase	6-Br-2-naphthyl-βD-glucopyranoside	5.4	-	-	-
N –acetyl - β - glucosaminidase	1-naphthyl-N-acetyl-βD-glucosaminide	5.4	-	-	-
α – mannosidase	6-Br-2-naphthyl-α D-mannopyranoside	5.4	-	-	-
α – fucosidase	2-naphthyl-αL-fucopyranoside	5.4	-	-	-

+, Positive; -, negative reactions
